# XEN45 Implant in Medically Controlled vs. Uncontrolled Eyes—Differential IOP Changes in Real-Life Conditions

**DOI:** 10.3390/jcm13123406

**Published:** 2024-06-11

**Authors:** Gemma Julio, Raquel Larena, Marta Mármol, Anna Soldevila, María Isabel Canut, Josip Pavan, Rafael I. Barraquer

**Affiliations:** 1 Centro de Oftalmología Barraquer, 08021 Barcelona, Spain; gemma.julio@barraquer.com (G.J.); raquel.larena@barraquer.com (R.L.); marmolm@barraquer.com (M.M.); annasoldevila94@gmail.com (A.S.); mcanut@oftalvist.es (M.I.C.); 2 Institut Universitari Barraquer, Universitat Autònoma de Catalunya, 08021 Barcelona, Spain; 3 Clínica Oftalvist, 08017 Barcelona, Spain; 4 Department of Ophthalmology, Dubrava University Hospital, 10000 Zagreb, Croatia; josip.pavan@zg.htnet.hr; 5 School of Medicine, Universitat Internacional de Catalunya, 08193 Barcelona, Spain

**Keywords:** primary open-angle glaucoma, XEN45 implant, intraocular pressure, survival analysis, minimally invasive glaucoma surgery, MIGS

## Abstract

**Background:** To assess intraocular pressure (IOP) changes and complications after XEN45 implants in medically controlled eyes (MCE) vs. medically uncontrolled eyes (MUE). **Methods:** A retrospective study, in a tertiary referral hospital, on mild-to-moderate primary open-angle glaucoma (POAG) cases under topical medication, including 32 eyes with IOP < 21 mmHg (MCE group) and 30 eyes with IOP ≥ 21 mmHg (MUE group). The success criteria using Kaplan–Meier analysis was IOP < 21 mmHg without medications (complete success) or fewer drugs than preoperatively (qualified success) at the last visit, without new surgery or unresolved hypotony. **Results:** No significant preoperative differences were found between the groups. The mean IOP was 15.6 ± 3.8 mmHg in MCE and 15.1 ± 4.1 mmHg in the MUE group (*p* > 0.05; Mann–Whitney test) at the end of the follow-up (mean of 26.1 ± 15.6 months and 28.3 ± 15.3 months, respectively) (*p* = 0.414, Mann–Whitney Test). The device caused a significant IOP reduction at 24 h in both groups. Thereafter, the MCE group significantly tended to increase IOP, recovering baseline values at 1 month and maintaining them until the end of the follow-up. In contrast, in the MUE group, the IOP values tended to be similar after the first reduction. No relevant complications and no significant differences between the groups in the survival analysis were found. **Conclusions:** XEN45 provided stable IOP control in both the MCE and MUE group without important complications in the medium term. The IOP increasing in the MCE group, after a prior decrease, led to restored baseline values 1 month after surgery. The homeostatic mechanism that causes the rise in the IOP to baseline values and its relationship with failure cases remains to be clarified.

## 1. Introduction

Topical–hypotensive medication is the classic first-treatment approach to primary open-angle glaucoma (POAG), but long-term topical treatments induce dry eyes and fibrosis, thus reducing the probability of success after glaucoma filtering surgery [1,2,3,4,5,6,7,8,9,10,11].

Minimally invasive glaucoma surgery (MIGS) devices have emerged as safer and less invasive than traditional surgeries, decreasing IOP and reducing the medication burden [12,13,14,15,16]. They have been used alone, with one or two devices, or combined with phacoemulsification surgery or mitomycin C [8,14,17,18,19,20,21]. The XEN45 Glaucoma gel stent (Allergan plc, Dublin, Ireland) is a soft cross-linked porcine collagen ab interno implant inserted from the anterior chamber into the subconjunctival space using a preloaded injector. The device reduces IOP by draining aqueous fluid from the anterior chamber into the subconjunctival space. 

Prior studies with the XEN45 implant have shown the durable and safe reductions of IOP, with and without concomitant cataract surgery, leading to a decrease in or complete elimination of medication [8,16,19,20,21,22,23,24,25,26,27,28,29,30,31]. These studies were focused on analyzing medically uncontrolled eyes (MUE), mixed with medically controlled eyes (MCE) or eyes without previous medication. To our knowledge, there are no studies that assess MCE alone.

The XEN45 mechanical drainage effect is expected to be similar in both groups, with higher IOP reductions in the group with the highest baseline values [8,15,16,32], that is, the MUE group. In addition, it is plausible to think that internal regulation activity also influences the eye response after the device’s implantation. Describing IOP changes in each group separately could improve the knowledge of this issue considering that MCE seems to have easier IOP control with drugs than MUE does, which may be due to a better homeostatic function in MCE. 

The main objective of this study is to analyze IOP changes over time after the XEN45 implant in the MCE group and compare them with IOP changes in MUE. The clinical effects and survival analysis of the XEN45 implant in both groups were also assessed to complete this study. 

## 2. Materials and Methods

### 2.1. Ethics Statements

We conducted a retrospective comparative study of patients with POAG visited at the Centro Oftalmología Barraquer between October 2016 and February 2022. The approval from our Institutional Review Board (CEIm of the Centro Oftalmología Barraquer) was obtained for the review of the patient’s clinical records and the study adhered to the tenets of the Declaration of Helsinki (IRB/ethics number: 210_XEN45, approval: 05/02/2024). 

### 2.2. Patients

This study included consecutive eyes with POAG which underwent XEN45 Gel-Stent implantation (Allergan, Dublin, Ireland) surgery, alone or in combination with cataract extraction. Inclusion criteria were subjects ≥ 18 years of age with mild-to-moderate POAG, with or without cataracts, and who had at least a follow-up period of 6 months if XEN45 did not fail. Patients were excluded if they were not currently using IOP-lowering topical medication or had corneal or other ocular surgeries within 6 months before enrollment in the study, any corneal abnormality preventing reliable applanation tonometry, or other circumstances that can alter the results.

The sample was divided into 2 groups: The MCE group, which included eyes with IOP < 21 mmHg, in which elimination of topical drugs was considered, after informed consent of the patient, due to either drug intolerance and/or programmed cataract surgery. The MUE group included eyes with IOP ≥ 21 mmHg despite the use of the maximum topical anti-glaucoma drugs and reduction of IOP was mandatory.

### 2.3. Data Collection

At the baseline visit, the ophthalmic examination included a review of their medical/ophthalmic history, IOP measurement using Goldmann applanation tonometry (Haag Streit, Switzerland), Humphrey visual field (Carl Zeiss Meditec, Dublin, CA, USA), and central corneal thickness using an ultrasonic pachymeter (OcuSCAN RxP; Alcon Laboratories, Fort Worth, TX, USA). 

Follow-up visits (on day 1, week 1, month 1, 2, 3, 6, and annually) included standard ophthalmic examination with IOP measurements. These time points allowed correct temporary sampling to obtain a reliable description of the IOP changes that lead to IOP control.

The number of needlings, ocular hypotensive medication, and other surgical procedures were recorded. Gonioscopy and anterior segment optical coherence tomography (Cassia SS-1000 OCT (Tomey, Nagoya, Japan)) to assess bleb filtration and XEN45 position were performed at the ophthalmologist’s discretion. 

### 2.4. XEN45 Implantation Technique

A single XEN45 implant (lumen diameter = 45 µm; length = 6 mm) was implanted in all the study eyes. All surgical procedures were performed under local anesthesia by one experienced surgeon (MIC).

After subconjunctival injection of 0.1 mL mitomycin C diluted to 0.2 mg/mL (mitomycin-C, Xalabarderfarma Laboratory, Barcelona, Spain) in the superotemporal quadrant at 8 mm posterior to the limbus, an inferotemporal corneal incision was made, and the anterior chamber was filled with viscoelastic. Using the pre-loaded 27-gauge injector, the XEN45 was implanted via an ab interno approach in the supranasal quadrant, using a standard technique [19,20,21,22,23,24,25,26,27,28,29,30,31,32,33,34]. The ideal position of the stent is 3 mm in length subconjunctivally, 2 mm in the sclera, and 1 mm in the anterior chamber. At the end of the surgery, the XEN45 implant was cautiously moved through the conjunctiva with a blunt instrument to ensure that it was freely mobile with no Tenon tissue blocking the lumen of the tube. A balanced solution was injected into the anterior chamber, creating a low diffuse bleb to confirm patency. After that, the viscoelastic was removed and the incisions hydrated. 

In those patients where cataract surgery was indicated, a standard phacoemulsification technique was performed and, after the intraocular lens was properly placed in the bag, XEN45 implantation was carried out as previously described. 

Also, IOP-lowering medications were discontinued at the time of surgery. The standard postoperative regimen consisted of a topical antibiotic for 1 week and a tapering topical steroid for 4 weeks. 

Needling was performed in those patients with confirmed bleb fibrosis, flat bleb, and/or elevated IOP by the same glaucoma surgeon (MIC), with a 27-gauge hypodermic needle bent in the subconjunctival space. Episcleral adhesions above and below the device were released by sweeping motions, taking care not to pull or damage the device. At the end of the puncture procedure, 0.1 mL of 5-fluorouracil (50 mg/mL) (Fluorouracil Accord; Accord Laboratory, Cork, Ireland) was injected into the subconjunctival space.

### 2.5. Sample Size 

It was determined that a sample size of 29 eyes was required to detect a mean IOP difference between timepoints of 2 mm Hg, with a type I error of 0.05 and a power of 90%, assuming a standard deviation of 3.8 mm Hg. 

It is worth mentioning that temporal sampling (1 day, 1 week, 1, 2, 3, 6, and 12 months, and annually) was exhaustively carried out in all the included cases to obtain reliable patterns of IOP change.

### 2.6. Statistical Analysis

After an exploratory analysis, the Shapiro–Wilk test was used to analyze the normality of the data distribution. 

To analyze IOP changes over time (intragroup comparisons), Friedman’s test, and Wilcoxon rank test (for pair comparisons) were carried out and Bonferroni correction was applied (*p* < 0.007). 

*t*-test or Mann–Whitney test was performed for numerical variables when benefits and risk effects were assessed (intergroup comparisons). 

Categorical variables were compared using a Chi-square test and Fisher’s exact test, as needed.

Kaplan–Meier was performed for survival analysis, with Log-rank for comparison between the two groups. Success criteria were maintained IOP under 21 mmHg (in the MCE group) or reduced IOP under 21 mm Hg (in the MUE group) without antiglaucoma medications (complete success) or with fewer drugs than before surgery (qualified success) at the end of the follow-up. Failure criteria in both groups were the inability to achieve success conditions, new surgery, or hypotony (IOP less than 6 mmHg) that did not resolve in 1 month. XEN45 relocation, needlings, or bleb revision were not considered failures.

According to survival analysis, the follow-up period was the time between the XEN45 implant and the last recorded visit with no signs of failure or the interval of time between the surgery and the failure.

A *p*-value < 0.05 was considered statistically significant. All statistical analyses were performed with SPSS software V22 (SPSS Inc., Chicago, IL, USA). 

## 3. Results

Seventy-two medical records were reviewed, and 10 cases were excluded: two eyes without previous medication, three eyes diagnosed with pseudoexfoliative glaucoma, one eye with previous corneal surgery, and four eyes with a lack of follow-up. Finally, 32 eyes of 24 patients were included in the MCE group and 30 eyes of 25 patients in the MUE group. Table 1 illustrates the demographic and preoperative characteristics of the two groups. 

No baseline significant differences were found between the groups regarding gender, age, or the number of topical drugs. See the comparative results in Table 1.

### 3.1. Intraoperative Data

No intraoperative complications were found, but three eyes in the MUE group and one eye in the MCE group required XEN45 relocation, and one eye underwent bleb revision (see details below). 

Twenty eyes (67%) underwent a XEN45 implant combined with cataract surgery in the MUE group and twenty-seven eyes (84%) in the MCE group, without significant differences between groups (*p* = 0.141, Fischer’s exact test). 

### 3.2. Postoperative Data

The mean follow-up was 28.3 ± 15.3 months (range: 0.23–57 months) in the MUE group and 26.1 ± 15.6 months (range: 6–53 months) in the MCE group without significant differences (*p* = 0.414, Mann–Whitney Test). It is worth mentioning that cases with a lower follow-up than 6 months were due to the presence of failure. 

**Figure 1:**
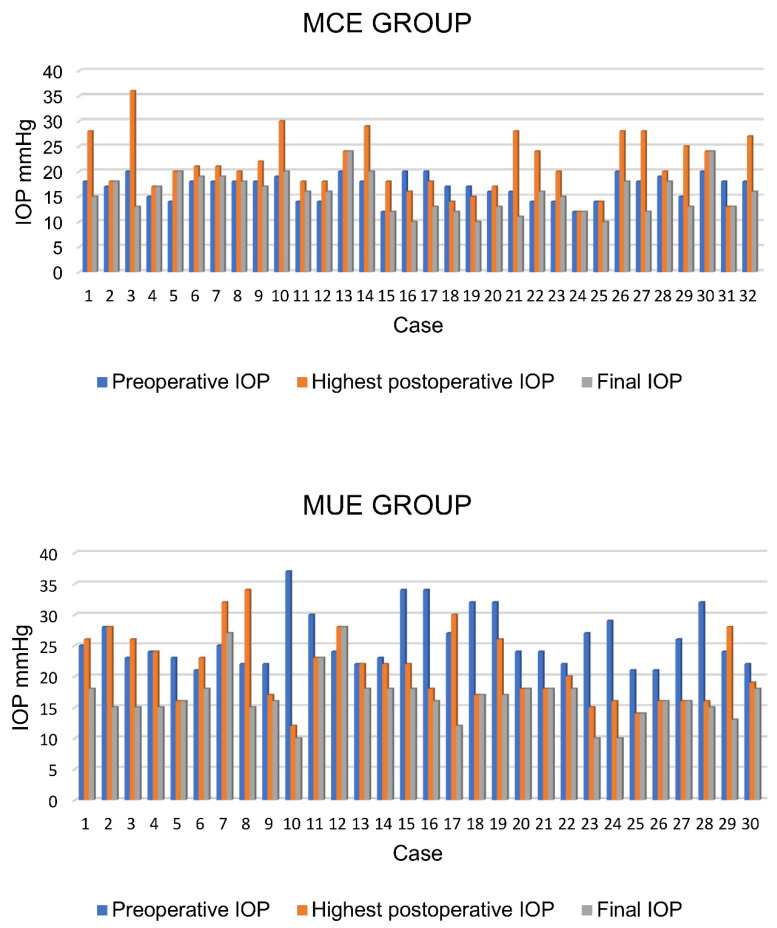
IOP changes in each eye.

**Figure 2:**
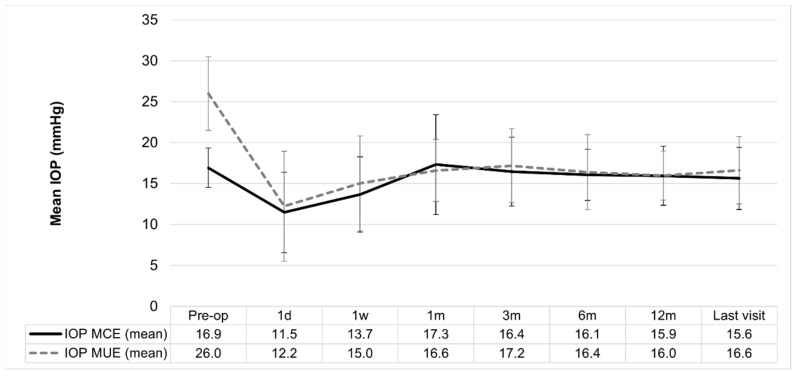
IOP changes during follow-up. Bars represent the standard deviation, respectively. Pre-op: baseline IOP; d: day; w: week; m: month.

At the end of the follow-up, 94% (30/32) of the cases in the MCE and 93% (28/30) in the MUE group had IOP values ≥ 6 and ≤21 mmHg. The mean IOP was 15.6 ± 3.8 mmHg in the MCE and 16.6 ± 4.1 mmHg in the MUE group without significant differences between the groups (*p* > 0.05; Mann–Whitney test). Figure 1 and Figure 2 illustrate individual IOP changes (see Table 2).

The mean number of final IOP-lowering medications was 0.6 ± 0.8 (median: 0 drugs; range: 0–2 drugs; IQR: 1 drug) in the MCE group and 0.6 ± 1.0 (median: 0 drugs; range: 0–3 drugs; IQR: 1.3 drugs) in the MUE group without significant differences between them (*p* = 0.5, Fischer’s Exact test).

**Figure 3:**
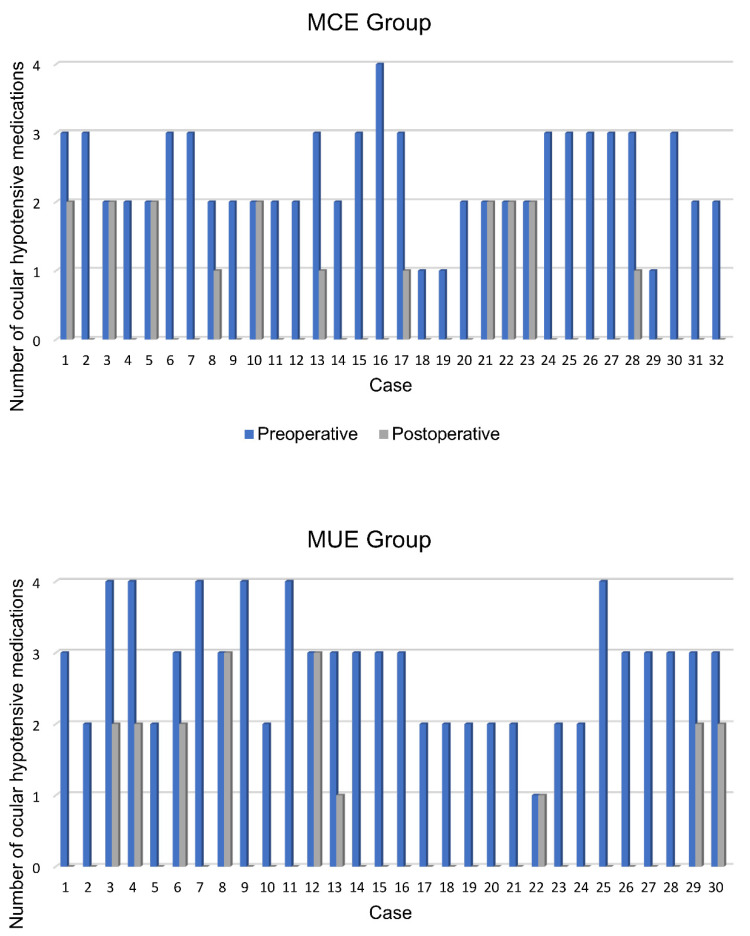
Changes in the number of ocular hypotensive topical medications in each patient.

Neither IOP-lowering medication nor new surgeries were present in 20/32 eyes (63%) in the MCE group and 20/30 (67%) in the MUE group. No cases with an increased number of medications were found (Figure 3 shows the changes in the number of topical medications in each patient).

In the MCE group, 7/32 eyes (22%) required eight needlings at different times after the XEN45 implant. In the MUE group, 7/30 eyes (23%) needed one needling, and one case required bleb revision at 43 months.

Postoperative complications in the MCE group were IOP peaks (IOP increased ≥ 10 mmHg or IOP ≥ 30 mmHg) in 8/32 eyes (25%), which were resolved in all cases (see Table 3), and self-limited hypotony in 5/32 eyes (16%) at day 1, with no macular alterations. Postoperative complications in the MUE group were IOP peaks in 3/30 (10%), resolved in all the cases, and self-limited hypotony in 5/30 (17%). See details in Table 3.

### 3.3. Intragroup IOP Changes over Time

In the MCE group, a significant IOP reduction (*p* = 0.001; Wilcoxon test) from the baseline was observed at 24 h (median difference of 5.5 mmHg; 95% CI: 3.5–7 mmHg) and 1 week after surgery (median difference of 3 mmHg; 95% CI: 1.5–5 mmHg). Interestingly, after this prior IOP reduction, there was an IOP increase, 1 month after surgery, and the group achieved similar values to the baseline until the end of the follow up. 

Regarding the MUE group, a significant IOP reduction from the preoperative values was observed 24 h after XEN45 implantation and it was maintained until the end of the follow-up (*p* = 0.001, Wilcoxon test, in each comparison with the baseline data). The median IOP reduction ranged from 14 mmHg (95% CI = 11 to 17 mmHg) at day one to 9.5 mmHg (95% CI = 6.5 to 11 mmHg) at the last visit. 

### 3.4. Survival Analysis

In the MCE group, the overall rate of complete success was 63% and four cases were classified as a qualified success (12%). One of the successful cases needed one needling. See Table 4 for individual details of the qualified success and failure.

For the MUE group, the overall rate of complete success was 63% and six cases were a qualified success (20%). Two eyes required one needling. Table 4 describes individual details of the qualified successes and failure cases.

**Figure 4:**
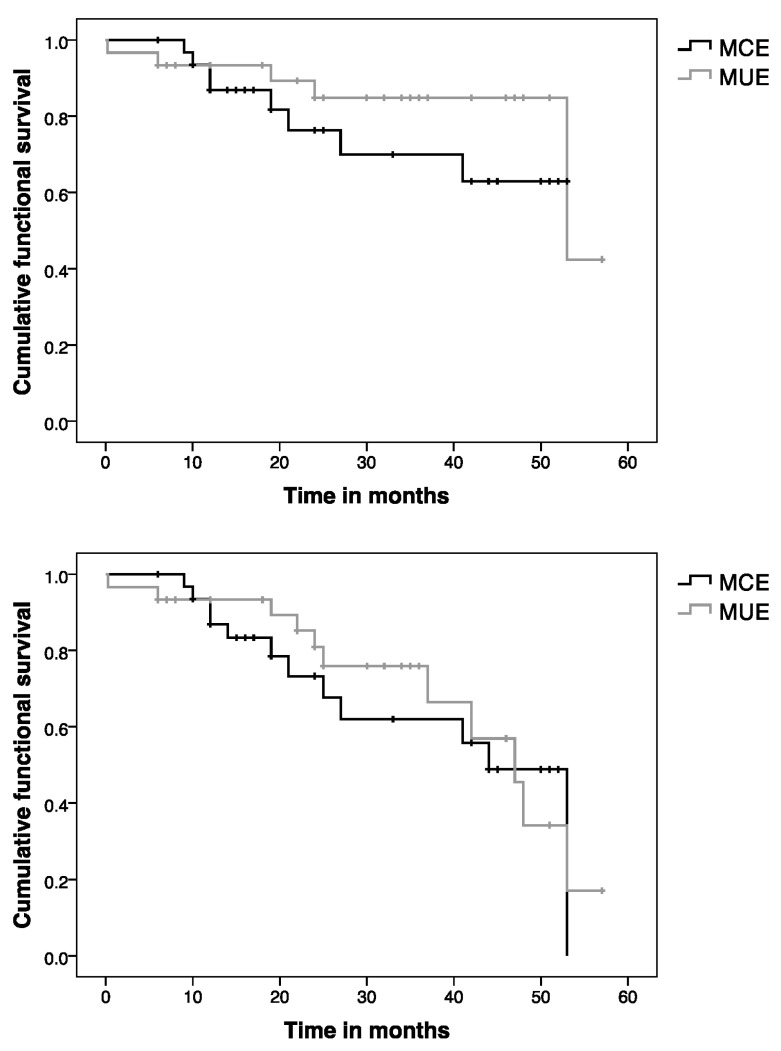
Survival analysis. Top: Survival analysis (success: complete and qualified success). Bottom: Survival analysis (success: only complete success). MCE: medically controlled eyes; MUE: medically uncontrolled eyes.

Applying Kaplan–Meier survival analysis (Figure 4), the cumulative probability of success (complete + qualified success) in the MCE group was 87%, 76%, and 63% at 1, 2, and 4 years, respectively, with a mean survival time of 42 ± 3.5 months (95% CI: 35–48 months). The cumulative probability of complete success was 87%, 73%, and 49% at 1, 2, and 4 years with a mean survival time of 38 ± 3.6 months (95% CI: 31–45 months) for the MUE group.

Regarding the MUE group, the cumulative probability of success was 93% at 1 year and 85% at 2 and 4 years after surgery with a mean survival time of 49 ± 3.2 months (95% CI: 43–55 months). The cumulative probability of complete success was 93%, 81%, and 34% at 1, 2, and 4 years with a mean survival time of 41 ± 3.3 months (95% CI: 35–45).

There were no significant differences between both survival curves considering all the successful cases (*p* = 0.324; Log-Rank) or only the complete successes (*p* = 0.704; Log Rank).

## 4. Discussion

These findings demonstrated the adequate effectiveness and safety of the XEN45 implant in the medium term to eliminate topical medication in both groups without significant complications. The device triggered significant IOP reduction at 24 h in both groups and, interestingly, 1 month after surgery, IOP behavior was different in each group. The MCE group tended to recover preoperative IOP values (that is, to increase values after this first reduction), while IOP reduction was maintained in the MUE group during all the follow-ups, thus achieving mean normal values. To our knowledge, it is the first time that these differential changes in IOP after the implant have been described. 

Developing a hypothesis, we modestly think that an IOP increase may be caused by an increase in the aqueous humor inflow, which leads MCE to baseline normal values, thus modulating the ‘excessive’ prior IOP reductions (excessively far from baseline values) after the XEN45 implant. 

Unfortunately, the knowledge of the intricate IOP homeostasis mechanisms has some light and shadows. Interestingly, it has been reported that modulating chronic inflammation in glaucoma by eliminating stress stimuli, such as increased intraocular pressure (IOP), long-term treatments, and the number of topical antiglaucoma eye drops, is crucial for achieving a physiological level of para-inflammation required for tissue homeostasis maintenance and functional restoration [33].

Nevertheless, knowing more about the influence of the homeostatic mechanisms on the implant’s effectiveness seems to be important, above all to explain failure cases. Indeed, a few eyes in the sample will not be able to internally regulate IOP, and this must be taken into consideration before implantation. The device may produce different results depending on the functionality of the homeostatic mechanisms, which weaken over time in glaucoma conditions [8,33]. If our hypothesis is confirmed in the future, we suggest, as with the new SLT evidence, to revise the disease management algorithm. Specifically, an evaluation of including MIGS therapies earlier could be interesting to achieve the maximum effectiveness of the implant, thus reducing the inflammation level that topical treatment involves. A further assessment will be necessary to resolve these questions. 

In all previous studies [19,22,23,24,25,26,27,28,29,30,31,34,35,36], medically controlled eyes were merged into the same group with eyes without a response to topical medication. Due to this, these studies presented methodological challenges during data analysis, such as establishing an activity indicator suitable for both situations, since some of the most frequently used criteria, such as the % of IOP reduction (Table 5), are not useful indicators of responses in MCE. 

Regarding the IOP cut-off value for success, we established a relatively high value (<21 mmHg) because the sample of eyes presented mild-to-moderate POAG with eye-drop treatment and no previous surgeries. Other studies have suggested lower cut-off values if the patients suffers advanced POAG and previous surgeries [20,21].

The analysis of controlled and uncontrolled eyes separately leads to a lesser dispersion of the results and more reliable conclusions, avoiding confounding factors and allowing the different ‘strategies’ of the internal regulatory system to be verified.

In addition, the implant effectiveness in both groups in terms of the mean final IOP and the number of topical medications was in the range of other published studies, although the comparison is difficult because, as previously mentioned, the samples were mixed (with MUE and MCE in the same group), and had several open-angle glaucoma etiologies or did not have specific information on the baseline glaucoma control (Table 5).

Regarding safety, no patients experienced serious complications related to conventional glaucoma procedures, such as endophthalmitis, and only three cases in the MUE (10%) and one in the MCE (3.1%) group needed stent repositioning, which is a percentage similar to that described in the literature (1–12.5%) [19,39,41]. Furthermore, we found a percentage of hypotony (16% in MCE and 13% in the MUE group) that was also within the range of values (8–20%) of other studies [27,35]. It is worth mentioning that the cases with IOP peaks and hypotony were completely resolved in subsequent weeks with no adverse events.

Some authors have described a sustained reduction in IOP in the early postoperative period (≤1 month) as a positive predictive factor of success [19,35]. Our results support this idea since the tendency was to reach the success conditions within 1 month after surgery and to maintain it during the follow-up. 

The number of eyes that underwent XEN45 implantation combined with cataract surgery was similar in both groups. Hence, the surgery type is not a confounding factor in the analysis. At the beginning of the use of MIGS, some authors suggested that combined surgeries could be a determining factor for success, but this association is still unclear [20,37,38,39,40,42,43,44,45]. The latest evidence using logistic regression in a cohort of 92 eyes found no relationship between success and combined surgery [43]. In addition, a recent systematic review reported that combined surgery seemed to have a higher rate of failure rather than standalone procedures, but no significant differences between standalone and combined procedures were found [20]. Nevertheless, some controversy still exists and further assessments are needed. In real-life clinical practice, when cataract surgery is needed, a combined procedure would be a good option to maintain or achieve IOP control with minimal additional interventions. 

Needlings were needed in 22% of the MCE eyes and 23% of the MUE eyes, which is fewer than in previously published reports (30–40%) [23,35,42]. It has been suggested that cases with lower IOP on day 1 need fewer needlings [44]. This is supported by Fea A.M. et al. 2020 [19], who found a significant correlation between needlings and IOP on day 1, week 1, and month 1. In our experience, ensuring the initial mobility and patency of the implant in the operating room is key to achieving the best results. However, different criteria for needling may be another reason for our lower percentage.

Among the limitations of the current study, its retrospective design is the most important one. Nevertheless, the inclusion/exclusion criteria minimized the inherent selection bias and confounding factors. 

The included eyes did not have a washout period prior to XEN45 implantation. It could be necessary to assess the effect of the device without previous drugs. However, in this study, the differences found in the IOP postoperative pattern were equally affected by the lack of a washout period, and the group comparison provided relative measurements not absolute ones. On the other hand, washout could alter the real-life IOP conditions that we wanted to analyze. 

In this study, some paired eyes from the same patient were included. This may affect the independence of the data, but glaucoma, although a bilateral disease, often shows asymmetric progression. Further clinical trials will be necessary to comprehensively assess the long-term role of MIGS and update the therapeutic algorithm for glaucoma management. 

## 5. Conclusions

XEN45 provided stable IOP control in the MCE and the MUE groups without important complications in the medium term. Interestingly, IOP increasing in the MCE group, after a prior decrease, led to the restoration of the baseline values 1 month after surgery. 

It remains to be clarified what the key mechanism is that increases the IOP to the baseline values. Further studies will be necessary to elucidate this question.

Knowing the differential pattern of response to the device is essential for the optimal treatment of glaucoma patients characterized by variable progressions and therapy responses.

## Figures and Tables

**Table 1:** Demographic, preoperative clinical conditions, and type of surgery in the studied groups.

	MCE Group (n = 32 Eyes)	MUE Group (n = 30 Eyes)	*p*-Value
Age, years			
Mean (SD)	68 ± 12	65 ± 13	0.407 ^a^
Min–Max	48–86	35–87	
Gender, n (%)			
Male	12 (50)	14 (56)	0.778 ^b^
Female	12 (50)	11 (44)	
IOP, mmHg			
Mean (SD)	16.9 ± 2.4	26.0 ± 4.5	0.001 ^c^
Min–Max	12–20	21–37	
Lens status, n (%)			
Phakic	28 (87.5)	23 (76.7)	0.395 ^b^
Pseudophakic	4 (12.5)	6 (20.0)	
Aphakic	0 (0)	1 (3.3)	
CCT, µm			
Mean (SD)	536.3 ± 39.9	527.9 ± 36.4	0.528 ^a^
Min–Max	435–609	449–628	
Number of AGM			
Mean (SD)	2.4 ± 0.7	2.8 ± 0.8	0.147 ^b^
Min–Max	1–4	1–4	
Median	2	3	
IQR	1	1	
Type of surgery, n (%)			
XEN	5 (16)	10 (33)	0.141 ^b^
XEN + Phaco	27 (84)	20 (67)	

AGM: Antiglaucoma Medication; CCT: Central Corneal Thickness; IQR: Interquartile range; Min: Minimum; Max: Maximum; SD: Standard deviation; MCE: medically controlled eyes; MUE: medically uncontrolled eyes; (^a^): *t*-test; (^b^): Fischer’s Exact test; (^c^): U-Mann–Whitney test.

**Table 2:** Mean IOP in each group during follow-up.

	MCE Group Mean ± SD Min–Max (mmHg)	MUE Group Mean ± SD Min–Max (mmHg)	*p*-Value
Preoperative	16.9 ± 2.4	26.0 ± 4.5	0.001 ^b^
	(12–20)	(21–37)	
Day 1	11.5 ± 4.9	12.2 ± 6.7	0.843 ^b^
	(2–25)	(4–32)	
Week 1	13.7 ± 4.6	15.0 ± 5.8	0.826 ^b^
	(4–22)	(8–30)	
Month 1	17.3 ± 6.1	17.3 ± 3.8	0.584 ^a^
	(8–36)	(10–23)	
Month 3	16.4 ± 4.2	17.2 ± 4.5	0.605 ^b^
	(11–30)	(10–28)	
Month 6	16.1 ± 3.14	16.4 ± 4.6	0.423 ^b^
	(10–24)	(10–34)	
Month 12	15.9–3.6	16.0 ± 3.0	0.647 ^b^
	(10–28)	(9–22)	
Last visit	15.6 ± 3.8	16.6–4.1	0.394 ^b^
	(10–24)	(10–28)	

Min: Minimum; Max: Maximum; SD: Standard deviation; MCE: medically controlled eyes; MUE: medically uncontrolled eyes; (^a^): *t*-test. (^b^): U-Mann–Whitney test.

**Table 3:** Postoperative complications in the sample. Hypotony and IOP peak cases in both groups.

	CC	Baseline IOP (mmHg)	IOP Spike or Hypotony (Time) *		Treatment	Survival (Time) in Months
MCE group						
	1	18	28 (m 12)		N (1)	QS (14)
	3	20	36 (m 1)		Corticosteroid cessation	F (21)
	4	15	5 (w 1)		-	S (12)
	10	19	30 (m 2 and 3)		N (2)	F (10)
	14	18	29 (m 3)		N (1)	S (15)
	17	20	5 (24 h)		-	QS (53)
	21	16	28 (m 38)		BB + CAI	F (41)
	22	14	24 (m 6)		BB + CAI	F (12)
	24	12	2 (24 h)		-	S (17)
	25	14	5 (24 h)		-	S (17)
	27	18	28 (m 1)		N (1)	S (45)
	29	15	25 (m 1)		Eye massage	S (24)
	31	18	4 (24 h)		-	S (10)
MUE group						
	38	21	4 (24 h)		-	QS (53)
	39	25	32 (24 h)		NPDS	F (0.23)
	40	22	34 (m 6)		BB + CAI	F (25)
	42	37	5 (24 h)		-	S (46)
	49	27	5 (24 h)	30 (w 1)	N (1)	S (51)
	55	27	4 (24 h)		-	S (30)
	57	21	4 (24 h)		-	S (22)

* Altered IOP was considered when IOP increase was ≥10 mmHg or IOP ≥ 30 mmHg; BB + CAI = beta-blocker and carbonic anhydrase inhibitor; CC = case code; m = months; N = Number of needling (n); NPDS = non-penetrating deep sclerectomy; m = month; QS = qualified success; S = success; F = failure; w = week. Note: Hypotony cases (IOP < 6 mmHg) are in white. All these cases were self-limited before 1 month; IOP peaks at 12 months or later are in dark grey, and IOP peaks before 12 months are highlighted in light grey.

**Table 4:** Failure and qualified cases’ description in both groups.

	CC	Survival Classification	Age (Years)	Baseline Ocular Pathologies	Baseline IOP (mmHg)	Previous AGM	Surgery	Needling Time (Months)	Final IOP (mmHg)	Final Topical Treatment	Survival Time * (Months)	Surgery after XEN Implant (Time in Months)
MCE group												
	1	QS	61	-	18	BB, PG, CAI	XEN	12	15	BB, PG	14	-
	8	QS	78	-	18	BB, PG	CEI + IOL + XEN	-	18	PG	44	-
	17	QS	62	-	20	BB, PG, CAI	CEI + IOL + XEN	-	13	BB	53	-
	28	QS	69	-	19	BB, CAI, AA	CEI + IOL + XEN	-	18	PG	25	-
	3	F	51	-	20	BB, CAI	XEN	-	**21**	BB, IAC	13	-
	5	F	73	HM	14	BB, PG	CEI + IOL + XEN	-	20	**BB, AA**	27	PPV (26)
	10	F	69	-	19	PG, CAI	CE + IOL + XEN	2 and 3	20	**PG, CAI**	10	-
	13	F	80	-	20	BB, CAI, AA	CE + IOL + XEN	-	**24**	BB	9	-
	21	F	86	-	16	BB, PG	CE + IOL + XEN	-	**11**	**BB, CAI**	41	
	22	F	63	CRVO	14	BB, PG	CE + IOL + XEN	-	16	**BB, CAI**	12	-
	23	F	63	-	14	BB, PG	CE + IOL + XEN	-	15	**BB, CAI**	12	-
	30	F	78	-	20	BB, PG, CAI	CE + IOL + XEN	-	24	**-**	19	**NPDS**
MUE group												-
	35	QS	87	-	23	BB, PG, CAI, AA	CE + IOL + XEN	-	15	BB, CAI	48	XEN_R (27)
	36	QS	87	-	24	BB, PG, CAI, AA	XEN	-	15	BB, CAI	47	-
	38	QS	60	HM	21	BB, PG, AA	CE + IOL + XEN	-	18	BB, CAI	53	-
	45	QS	60	-	22	BB, PG, AA	CEI + IOL + XEN	3	18	BB	42	-
	61	QS	69	-	24	BB, PG, AA	CEI + IOL + XEN	0.23	13	PG, AA	37	-
	62	QS	78	-	22	BB, PG, CAI	CE + IOL + XEN	-	18	BB, CAI	15	-
	39	F	61	CNVM + HM + FFS	25	BB, PG, CAI, AA	XEN	-	27	-	0.23	**NPDS + XEN_R**
	40	F	82	RD	22	BB, IAC, AA	XEN	1	15	BB, PG, CAI	25	-
	43	F	60	CC	30	BB, PG, CAI, AA	XEN	-	**23**	-	19	-
	44	F	35	-	24	BB, PG, CAI	XEN	3	28	BB, PG, CAI	6	**NPDS**
	54	F	72	HM	22	BB	CE + IOL + XEN	-	18	**PG**	24	-

AA: Alpha-adrenergic; BB: Beta-blockers; CAI: Carbonic anhydrase inhibitor; CC: Congenital cataract; CE: Cataract extraction; CC = case code; CNVM: Choroidal neovascular membrane; CRVO: Central retinal vein occlusion; F: Failed; FFS: Foster’s Fuchs Spot; HM: High myopia; IOL: Intraocular lens; MCE: medically controlled eyes; MUE: medically uncontrolled eyes; NPDS: Non-penetrating deep sclerectomy; PG: Prostaglandin; QS: Qualified success; PPV: Pars plana vitrectomy; RD: Retinal detachment; XEN_R: XEN repositioning; * Time until failure or last visit, depending on survival classification. The grey cells are a failure cause.

**Table 5:** Comparative mean final IOP and medication between the MCE and the MUE group with other published studies.

		Preoperative Mean IOP (mmHg)	Postoperative Mean IOP (mmHg)	% IOP Reduction	Mean Preop Drugs	Mean Postop Drugs	% Medication Reduction
Present study	Glaucoma control						
	MCE group Last visit	16.9 ± 2.4	15.6 ± 3.8	-	2.4 ± 0.7	0.6 ± 0.8	73.9
	MUE group Last visit	26 ± 4.5	16.6 ± 4.1	36.2	2.8 ± 0.8	0.6 ± 1	78.6
Other authors							
Galal et al., 2017 [37]	MUE and MCE	16 ± 4	12 ± 3	23	1.9 ± 1	0.3 ± 1	95
Grover et al., 2017 [38]	MUE	25.1 ± 3.7	15.9 ± 5.2	35.6	3.5 ± 1	1.7 ± 1.5	38.5
De Gregorio et al., 2018 [18]	Not specified	22.5 ± 3.7	13.1 ± 2.4	41.8	2.5 ± 0.9	0.4 ± 0.8	84
Mansouri et al., 2018 [39]	MUE	20 ± 7.1	13.9 ± 4.3	31	1.9 ± 1.3	0.5 ± 0.8	73.7
Heidinger et al., 2019 [27]	Not specified	22.8 ± 6.9	17.1 ± 5.9	22.7	2.9 ± 1	1.8 ± 1.4	37.9
Karimi et al., 2019 [31]	Not specified	19.3 ± 6.0	14.2 ± 4.4	25.3	2.6 ± 1.1	0.8 ± 1	69.2
Reitsamer et al., 2019 [40]	MUE	21.4 ± 3.6	14.9 ± 4.5	30.4	2.7 ± 0.9	0.9 ± 1.1	66.7
Fea et al., 2020 [19]	MUE and MCE	23.9 ± 7.6	15.5 ± 3.9	35.2	3 ± 1.1	0.5 ± 1	83.3
Gabbay et al., 2020 [23]	Not specified	22.1 ± 6.5	15.4 ± 5.9	30.5	2.77 ± 1.1	0.3 ± 0.7	-
Ventura-Abreu et al., ^♣^ 2020 [35]	MUE	23.5 ± 6.9	16.9 ± 3.8	31.2	2.3 ± 1.1	1.2 ± 1.5	-
Ibáñez-Muñoz et al., ^¥^ 2020 [24]	MUE	22.8	15.4	28.7	3.6	1.3	-

IOP: Intraocular pressure; ^¥^ only POAG results of the study; ^♣^ according to Figure 1 of the study. MCE: medically controlled eyes; MUE: medically uncontrolled eyes.

## Data Availability

The datasets used and/or analyzed during the current study are available from the corresponding author on reasonable request.
